# Highly Prevalent *LIPH* Founder Mutations Causing Autosomal Recessive Woolly Hair/Hypotrichosis in Japan and the Genotype/Phenotype Correlations

**DOI:** 10.1371/journal.pone.0089261

**Published:** 2014-02-19

**Authors:** Kana Tanahashi, Kazumitsu Sugiura, Michihiro Kono, Hiromichi Takama, Nobuyuki Hamajima, Masashi Akiyama

**Affiliations:** 1 Department of Dermatology, Nagoya University Graduate School of Medicine, Nagoya, Japan; 2 Takama Dermatology Clinic, Kasugai, Japan; 3 Department of Healthcare Administration, Nagoya University Graduate School of Medicine, Nagoya, Japan; Innsbruck Medical University, Austria

## Abstract

Mutations in *LIPH* cause of autosomal recessive woolly hair/hypotrichosis (ARWH), and the 2 missense mutations c.736T>A (p.Cys246Ser) and c.742C>A (p.His248Asn) are considered prevalent founder mutations for ARWH in the Japanese population. To reveal genotype/phenotype correlations in ARWH cases in Japan and the haplotypes in 14 Japanese patients from 14 unrelated Japanese families. 13 patients had woolly hair, and 1 patient had complete baldness since birth. An *LIPH* mutation search revealed homozygous c.736T>A mutations in 10 of the patients. Compound heterozygous c.736T>A and c.742C>A mutations were found in 3 of the patients, and homozygous c.742C>A mutation in 1 patient. The phenotype of mild hypotrichosis with woolly hair was restricted to the patients with the homozygous c.736T>A mutation. The severe phenotype of complete baldness was seen in only 1 patient with homozygous c.742C>A. Haplotype analysis revealed that the alleles containing the *LIPH* c.736T>A mutation had a haplotype identical to that reported previously, although 4 alleles out of 5 chromosomes containing the *LIPH* c.742C>A mutation had a different haplotype from the previously reported founder allele. These alleles with c.742C>A are thought to be the third founder *LIPH* mutation causing ARWH. To accurately determine the prevalence of the founder mutations, we investigated allele frequencies of those mutations in 819 Japanese controls. Heterozygous c.736T>A mutations were found in 13 controls (allele frequency: 0.0079; carrier rate: 0.016), and heterozygous c.742C>A mutations were found in 2 controls (allele frequency: 0.0012; carrier rate: 0.0024). In conclusion, this study confirms the more accurate allele frequencies of the pathogenic founder mutations of *LIPH* and shows that there is a third founder mutation in Japan. In addition, the present findings suggest that the mutation patterns of *LIPH* might be associated with hypotrichosis severity in ARWH.

## Introduction

Autosomal recessive woolly hair/hypotrichosis (ARWH: OMIM #278150/604379) is a rare hereditary hair disease characterized by tightly curled hair at birth. It can lead to sparse hair later in life. The disease was shown to be caused by mutations in *LIPH* or *LPA*R6 [1], [2]. The *LIPH* gene encodes a membrane-associated phosphatidic acid-preferring phospholipase A_1_α, which produces lysophosphatidic acid from phosphatidic acid and plays a crucial role in hair growth in humans [2]–[4].

The 2 *LIPH* mutations c.736T>A and c.742C>A have been reported as extremely prevalent causative mutations for ARWH in the Japanese population. 2 patterns of *LIPH* mutation–homozygous c.736T>A, and compound heterozygous c.736T>A and c.742C>A–are particularly frequent [5]–[9]. The *LIPH* mutations c.736T>A (p.Cys246Ser) and c.742C>A (p.His248Asn) were proven to be dysfunctional by *in vitro* studies [6]. The frequencies of the c.736T>A and c.742C>A alleles in healthy Japanese control individuals are 1.5% (3/200) and 0.5% (1/200), respectively, which implies the existence of many carriers for each mutation in the Japanese population [6].

Haplotype analysis in a previous report [6] found the chromosome containing the *LIPH* c.736T>A mutation to have 1 haplotype (ATCAACCGGA) and the haplotype of the chromosome containing the *LIPH* c.742C>A mutation to have the other haplotype (GCTCGTGAGG). This revealed that the missense mutations c.736T>A (p.Cys246Ser) and c.742C>A (p.His248Asn) in Japanese patients appear to represent founder effects in this island nation [6].

We recently reported 5 cases of ARWH and presented on 1 patient with mild hypotrichosis with the homozygous mutation c.736T>A [9]. To more accurately confirm the allele frequencies of the 2 founder mutations in Japan and to reveal genotype/phenotype correlations of ARWH, we have analyzed the clinical features, molecular basis and complete haplotype of 14 Japanese ARWH patients and analyzed the founder mutations with 819 Japanese control individuals.

## Materials and Methods

### Subjects

819 Japanese controls were recruited [10]. 14 unrelated, non-consanguineous Japanese families with ARWH were seen in our hospital or referred to us in the previous 2 years. 5 patients (Patients 1, 4, 5, 6 and 11) were previously presented [9]. Clinical information of the patients is summarized in Table 1. The Medical Ethics Committee of Nagoya University approved all the described studies, which were conducted according to the Declaration of Helsinki Principles (#1088-5). The participants gave written informed consent. Written informed consent was obtained from the guardians on behalf of the children enrolled. We recorded participant consent in paper. The ethics committees approved this consent procedure.

**Table 1:** Mutations in the *LIPH* gene in 14 families.

patient	age	sex	family	hypotrichosis	severity	*LIPH* mutation	father	mother	sibling
1	25	F	A	(+)	mild	c.736 T>A homo	N.A.	c.736T>A hetero	
2	40	F	B	(+)	mild	c.736 T>A homo	N.A.	N.A.	
3	34	F	C	(+)	mild	c.736 T>A homo	N.A.	N.A.	
4	27	F	D	(+)	severe	c.736 T>A homo	N.A.	N.A.	
5	35	F	E	(+)	severe	c.736 T>A homo	N.A.	N.A.	
6	3	M	F	(+)	severe	c.736 T>A homo	c.736T>A hetero	c.736T>A hetero	c.736T>A hetero
7	7	F	G	(+)	severe	c.736 T>A homo	N.A.	c.736T>A hetero	
8	12	F	H	(+)	severe	c.736 T>A homo	c.736T>A hetero	c.736T>A hetero	c.736T>A hetero
9	5	F	I	(+)	severe	c.736 T>A homo	c.736T>A hetero	c.736T>A hetero	
10	20	M	J	(+)	severe	c.736 T>A homo	N.A.	c.736T>A hetero	
11	4	M	K	(+)	severe	c.736T>A, c.742C>A C.H.	c.736T>A hetero	c.742C>A hetero	
12	5	F	L	(+)	severe	c.736T>A, c.742C>A C.H.	c.736T>A hetero	c.742C>A hetero	c.736T>A hetero
13	7	F	M	(+)	severe	c.736T>A, c.742C>A C.H.	c.736T>A hetero	c.742C>A hetero	no mutation
14	70	F	N	(+)	very severe	c.742C>A homo	N.A.	N.A.	

N.A.: not analyzed, C.H.: compound heterozygote.

### Mutation Detection

*LIPH* mutation search was performed as previously reported [6]. Briefly, genomic DNA (gDNA) isolated from peripheral blood was subjected to polymerase chain reaction (PCR) amplification, followed by direct automated sequencing using an ABI PRISM 3100 Genetic Analyzer (Advanced Biotechnologies, Columbia, MD, USA). The entire coding regions of *LIPH*, including the exon/intron boundaries, were sequenced using gDNA samples from patients and their family members.

### Haplotype Analysis

We performed haplotyping by using 10 tag-single-nucleotide polymorphism (SNP) analysis with reference to a previous report [6]. The oligonucleotide primers were designed using the CLC Main Workbench software application (CLC Bio, Aarhus, Denmark).

### Genotyping of the *LIPH* c.736T>A and c.742C>A Mutations in the 819 Japanese Control Individuals

Genomic DNA was extracted from whole blood using the QIAamp DNA Blood Maxi Kit (Qiagen, Valencia, CA). Real-time PCR-based genotyping of the *LIPH* c.736T>A and c.742C>A mutations was performed with TaqMan™ MGB probe genotyping assay according to the manufacturer’s instructions (Roche Diagnostics, Basel, Switzerland). To detect an allele of each mutation, a set of 2 TaqMan™ MGB probes labeled with a fluorescent dye (FAM or VIC) and a quencher dye (non-fluorescent dye; NFD) followed by minor groove binder (MGB), and sequence-specific forward and reverse primers were synthesized by Life Technologies Corp. The sequences of the assay probes/primers are shown in Table 2 of this article’s supplementary material. Real-time PCR was performed with LightCycler 480 system II 384 plate (Roche Diagnostics) in a final volume of 5 µl containing 2x LightCycler 480 Probes Master (Roche Diagnostics), 200 nM probes for wild type and mutant each and 900 nM forward and reverse primers each, with 5 ng genomic DNA as the template. The thermal conditions were the following: 95°C for 10 min, followed by 45 cycles of 95°C for 10 s, 60°C for 60 s and 72°C for 1 s, with a final cooling at 40°C for 30 s. Endpoint fluorescence was measured for each sample well. Afterward, genotyping was performed using endpoint genotyping analysis in LightCycler 480 software. 819 Japanese controls were analyzed with the real-time PCR-based genotyping of *LIPH* mutations.

**Table 2:** Sequences of assay primers/probes.

Mutation	Primer/Probe	Sequence
c.736T>A	LIPH-736.f	CTCTTTCATCACTTCTTTTACAACCAA
	LIPH-736.r	GCCATTCCTATAATCCTGGTAGGA
	LIPH-736.probe(T)	VIC-TTCAGTATTTTAAA**T**GTGACCACC-NFD-MGB
	LIPH-736.probe(A)	FAM-ATTTCAGTATTTTAAA**A**GTGACCACC-NFD-MGB
c.742C>A	LIPH-742.f	ATCACTTCTTTTACAACCAAGGATTTC
	LIPH-742.r	CTCTCAGGGAAGACAGGTACAGGTATAC
	LIPH-742.probe(C)	VIC- TTAAATGTGAC**C**ACCAGAGGT-NFD-MGB
	LIPH-742.probe(A)	FAM-ATTTAAATGTGAC**A**ACCAGAGGT-NFD-MGB

## Results

### Clinical Features of the Patients

**Figure 1:**
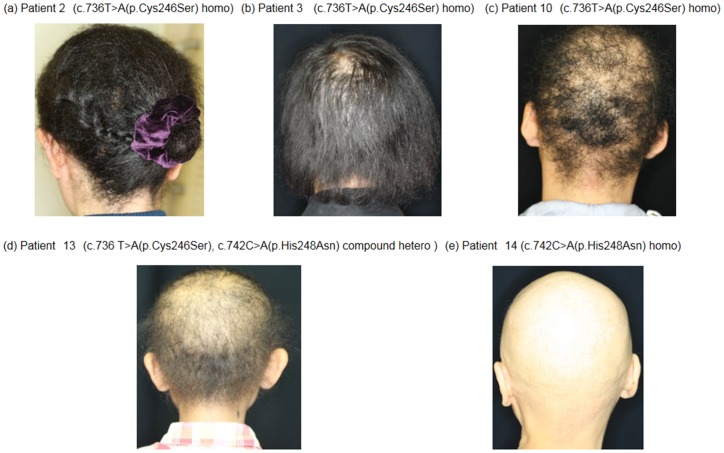
Clinical features of 5 Japanese families with ARWH. (a) Patient 2. (b) Patient 3. (c) Patient 10. (d) Patient 13. (e) Patient 14. All the affected individuals have features of ARWH, which is characterized by woolly hair on the scalp. Patient 2, with homozygous c.736T>A mutations. The hypotrichosis is notably mild, and the hair is longer than in the other patients. Patient 14, with homozygous c.742C>A mutations, has complete baldness. There were no notable differences in clinical features in patients other than these 4 cases.

13 of the patients had woolly hair and 1 of the patients had complete baldness since birth. 10 of the patients had only sparse scalp hair. 3 patients from Families A, B and C had mild hypotrichosis with woolly hair, and 1 patient from Family N had complete baldness (Table 1, Fig. 1).

### *LIPH* Mutation Detection in the Families with ARWH

Direct-sequencing analysis of exons and intron-exon boundaries of *LIPH* revealed that affected members of 10 families were homozygous for c.736T>A (p.Cys246Ser), and those of 3 families were compound heterozygous for the 2 missense mutations c.736T>A (p.Cys246Ser) and c.742C>A (p.His248Asn), and 1 patient from the other family was homozygous c.742C>A (p.His248Asn). All the patients’ parents whose DNA was available for mutation search were found to be heterozygous carriers of 1 or the other of the 2 mutations (Table 1).

### Genotype/phenotype Correlations in ARWH Patients with *LIPH* Founder Mutations

10 of the patients showed moderately severe ARWH phenotype, having only sparse scalp hair. 7 of these patients were homozygous for the c.736T>A mutation and the other 3 patients were compound heterozygous for the c.736T>A mutation and the c.742C>A mutation. 3 patients with homozygous c.736T>A mutations from Families A, B and C (see below) had mild hypotrichosis with woolly hair, and 1 patient with homozygous c.742C>A from Family N (see below) had the severe phenotype of complete baldness (Table 1, Fig. 1).

### Haplotype Analysis

The chromosome containing the *LIPH* c.736T>A mutation in all the patients and heterozygous carriers from all the families with the mutation c.736T>A had an identical haplotype (ATCAACCGGA). This haplotype was the same haplotype as that previously reported in the families with the *LIPH* mutation c.736T>A [6]. In all 4 families with the *LIPH* c.742C>A mutation, an identical haplotype (GCTCATGAGG) was detected in 4 out of 5 alleles containing the *LIPH* c.742C>A mutation, although the other allele had the haplotype (GCTCGTGAGG), which was previously reported in the founder mutation of c.742C>A [6]. It is known that the haplotype (GCTCATGAGG) is seen in less than 1% of the Japanese population [6]. Thus, the c.742C>A mutant alleles with the haplotype appear to be a third prevalent mutant *LIPH* allele among ARWH patients in this island nation.

### Screening for the Founder Mutations in Control Individuals

TaqMan™ MGB probe genotyping assay of *LIPH* revealed 13 out of 1638 (0.79%) alleles to have c.736T>A, and 2 out of 1638 alleles (0.12%) to have c.742C>A (Table 3). There were no controls that had homozygous or compound heterozygous mutations. Thus, the carrier rate for each mutation is 1.6% for c.736T>A and 0.24% for c.742C>A (Table 3).

**Table 3:** Allele frequencies of the founder mutation in 819 Japanese controls.

mutation	number of alleles in 819 Japanese controls	allele frequency	carrier rate
c.736T>A heterozygote	13/1638	0.79%	1.60%
c.742C>A heterozygote	2/1638	0.12%	0.24%

## Discussion

We analyzed 14 cases, including 7 adults, from 14 unrelated families with ARWH. All 14 cases were homozygous or compound heterozygous for the 2 prevalent *LIPH* mutations, c.736T>A and c.742C>A, in the Japanese population. Previous reports [5], [6], [8], [9] found all Japanese ARWH cases to be caused by homozygous c.736T>A mutations or compound heterozygous c.736T>A and c.742C>A mutations of *LIPH*. However, in our study, 1 case (Patient 14) had a homozygous c.742C>A mutation of *LIPH*. The parents of Patient 14 were cousins, while the other 13 families had non- consanguineous parents.

We analyzed haplotypes in these cases. In the previous report [6], alleles containing the *LIPH* mutations had only 2 haplotypes; the alleles containing the mutation c.736T>A had 1 identical haplotype (ATCAACCGGA), and the chromosome containing the mutation c.742C>A had another identical haplotype (GCTCGTGAGG). Those results revealed that Japanese patients appear to represent founder effects in this island nation. Interestingly, however, 4 out of the 5 alleles carrying the *LIPH* c.742C>A mutation had 1 different haplotype (GCTCATGAGG) in the 4 families with c.742C>A in the present study (Table 4). These 4 families were all from Nagoya and its environs, the central part of Japan’s main island. This result suggests that another founder containing c.742C>A exists in *LIPH* mutations of the Japanese population.

**Table 4:** SNPs for haplotype analysis with the *LIPH* c.736T>A and c.742C>A mutation.

Family	Mutation	SNP1	SNP2	SNP3	SNP4	SNP5	SNP6	SNP7	SNP8	SNP9	SNP10
A(Patient 1)	c.736T>A homo	A	T	C	A	A	C	C	G	G	A
B(Patient 2)	c.736T>A homo	A	T	C	A	A	C	C	G	G	A
C(Patient 3)	c.736T>A homo	A	T	C	A	A	C	C	G	G	A
D(Patient 4)	c.736T>A homo	A	T	C	A	A	C	C	G	G	A
E(Patient 5)	c.736T>A homo	A	T	C	A	A	C	C	G	G	A
F(Patient 6)	c.736T>A homo	A	T	C	A	A	C	C	G	G	A
G(Patient 7)	c.736T>A homo	A	T	C	A	A	C	C	G	G	A
H(Patient 8)	c.736T>A homo	A	T	C	A	A	C	C	G	G	A
I(Patient 9)	c.736T>A homo	A	T	C	A	A	C	C	G	G	A
J(Patient 10)	c.736T>A homo	A	T	C	A	A	C	C	G	G	A
K(Patient 11)	c.736 T>A, c.742C>A compound hetero	A/G	T/C	C/T	A/C	**A**	C/T	C/G	G/A	G	A/G
L(Patient 12)	c.736 T>A, c.742C>A compound hetero	A/G	T/C	C/T	A/C	**A**	C/T	C/G	G/A	G	A/G
M(Patient 13)	c.736 T>A, c.742C>A compound hetero	A/G	T/C	C/T	A/C	**A**	C/T	C/G	G/A	G	A/G
N(Patient 14)	c.742C>A homo	G	C	T	C	**A**/G	T	G	A	G	G

SNP1: rs6788865, SNP2: rs7615714, SNP3: rs12233604, SNP4: rs12233487, SNP5: rs12233490.

SNP6: rs12233622, SNP7: rs12233623, SNP8: rs1837882, SNP9: rs9790230, SNP10: rs497680.

We found 3 different severity levels of hypotrichosis among our cases. Patient 14 with the homozygous c.742C>A mutation of *LIPH* had the most serious baldness (Fig. 1). She reported having had complete baldness from childhood. In contrast, 3 females with the homozygous c.736T>A mutation (Patients 1–3) had long hair with mild hypotrichosis, apparently different from the clinical features of the other adult cases (Fig. 1). Patient 1 had had hypotrichosis in childhood, but it had improved in adulthood. She had topically applied commercially available 1% minoxidil solution to the scalp by herself until her first visit to our hospital, although it was not certain whether the minoxidil contributed to the improvement of her hypotrichosis. Patient 2 and Patient 3 showed curly hair, but hypotrichosis was very mild from birth without treatment. Except for these 4 cases (1 severe and 3 mild), the 10 other patients showed uniformly moderate clinical features (Fig. 1). Previous reports showed several *LIPH* mutation patterns to correlate with the severity of hypotrichosis in ARWH [11], [12]. However, there have been no reports on genotype/phenotype correlations between the 2 Japanese recurrent missense mutations c.736T>A (p.Cys246Ser) and c.742C>A (p.His248Asn) of *LIPH* and the severity of hypotrichosis. From the present findings, the mild phenotype might be restricted to homozygotes of mutation c.736T>A, and the homozygosity of c.742C>A might be associated with the severe phenotype. His248, but not Cys246, is thought to be a catalytic triad of LIPH [6]. Thus, c.742C>A (p.His248Asn) may affect the enzyme function more seriously than c.736T>A (p.Cys246Ser) does, although c.736T>A (p.Cys246Ser) and c.742C>A (p.His248Asn) were proven to affect the enzyme function to almost the same extent by *in vitro* studies [6]. We cannot exclude the possibility that the homozygous missense mutation in one of the catalytic triads, p.His248Asn, is related to the severe phenotype.

In this study, we determined allele frequencies of the 2 founder mutations in 819 controls. The frequencies of the c.736T>A and c.742C>A alleles in healthy Japanese control individuals are 0.79% (13/1638) and 0.12% (2/1638), respectively. We previously reported the frequencies of the c.736T>A and c.742C>A alleles in healthy Japanese control individuals to be 1.5% (3/200) and 0.5% (1/200), respectively [6]. There are no statistically significant differences between the 2 studies for each mutation. Combining the previous study and the present study, the allele frequencies of c.736T>A and c.742C>A are 0.87% (16/1838) and 0.16% (3/1838), respectively, and the carrier rates are 1.7% (16/919) and 0.32% (3/919), respectively. This suggests the existence of many carriers for each mutation in this island nation, especially for c.736T>A. The Ensembl genome browser (http://asia.ensembl.org/index.html) indicates that c.736T>A has not been found in any European, American, African or Asian population. The browser shows that c.742C>A has not been found in any European, American or African population, but has been found in Chinese populations. In Chinese populations, one out of 194 alleles is reported to be c.742C>A. These data suggest that c.742C>A might be a prevalent pathogenic mutation also in the Chinese.

## Conclusion

In conclusion, analysis of 14 unrelated families with ARWH confirmed that the 2 *LIPH* mutations c.736T>A and c.742C>A are apparently prevalent causative mutations for ARWH in the Japanese population. We found a novel founder haplotype of the ARWH-causing *LIPH* mutation. This founder is the second founder containing c.742C>A and the third founder for *LIPH* mutations. Interestingly, the present results suggested a genotype/phenotype correlation in *LIPH* mutations among ARWH patients. ARWH cases homozygous for c.742C>A showed a severe phenotype. In contrast, patients with mild hypotrichosis were seen among the ARWH patients homozygous for c.736T>A. Further accumulation of cases with *LIPH* mutations is needed to establish these genotype/phenotype correlations in *LIPH* mutations.

In addition, the present study confirmed the high frequencies of the 2 founder mutations, especially of c.736T>A, in 819 Japanese controls. Carrier rates of c.736T>A and c.742C>A are 1.7% and 0.32% respectively.
